# Qici Sanling decoction suppresses bladder cancer growth by inhibiting the Wnt/Β-catenin pathway

**DOI:** 10.1080/13880209.2019.1626449

**Published:** 2019-08-10

**Authors:** Hua Gong, Weihua Chen, Lanhua Mi, Dan Wang, Youkang Zhao, Chao Yu, Aiguang Zhao

**Affiliations:** a Department of Urology, Longhua Hospital, Shanghai, China;; b Department of Urology, Shanghai East Hospital, Shanghai, China;; c Department of Oncology, Longhua Hospital, Shanghai, China

**Keywords:** QCSL, TCM, XAV-939

## Abstract

**Context:** Bladder cancer, which has high recurrence, is one of the most deadly cancers in the world. *Astragalus propinquus* Schischkin (Fabaceae) and *Sagittaria sagittifolia* L. (Alismataceae) are important herbs reported to be effective in cancer therapy.

**Objective:** The efficacy of QCSL (Qici Sanling decoction) in bladder cancer treatment was examined.

**Materials and methods:** T24 cells were injected into the flanks of nude mice and the mice were randomly divided into five groups: control; 20 mg/kg XAV-939 (an inhibitor of the WNT/β-catenin pathway); QCSL (100, 200, or 400 mg/kg). After 7 weeks, the mice were anaesthetised using isoflurane and the xenografts were excised to perform further experiments.

**Results:** Both XAV-939 (tumour volume: 379.67 ± 159.92 mm^3^) and QCSL (796.18 ± 101.6 mm^3^) dramatically suppressed tumour growth comparing with control group (3612.12 ± 575.03 mm^3^). XAV-939 and QCSL treatments decreased cell proliferation from 56.3 ± 0.05% to 29.02 ± 0.07% and 37.51 ± 0.04%, respectively. In agreement, more infiltration of immune cells and pyknotic cells upon XAV-939 (apoptosis rates: 43.92 ± 0.03%) and QCSL (34.57 ± 0.04%) treatment comparing with control group (15.59 ± 0.03%) were observed. Furthermore, TUNEL staining of xenograft tumours illustrated more apoptotic cells upon XAV-939 and QCSL treatment. Mechanistically, XAV-939 and QCSL treatments significantly inhibited WNT/β-catenin pathway in T24 xenograft tumours.

**Discussion and conclusions:** Our findings give new insights into the role of QCSL in bladder cancer and explore potential mechanisms contributing to the therapeutic effects of QCSL in bladder cancer.

## Introduction

Bladder cancer is a common tumour. According to a recent report (Antoni et al. 2017), there were 430,000 patients worldwide in 2012. The incidence of bladder cancer is almost 4 times more in men than in women. As reported, it is the fourth most common cancer in men and the 13th in women in the UK (McBride 2015). Smoking habits and exposure to carcinogens may result in the difference in incidence between sexes (Castelao et al. 2001). Superficial bladder cancer, which accounts for almost 80% of bladder cancer cases, is the main type of bladder cancer (Heney et al. 1983). The standard treatment for superficial bladder cancer is a combined treatment of Transurethral Resection of Bladder Tumour (TURBT) and adjuvant intravesical therapy including chemotherapy or vaccine-based therapy in the bladder (Cindolo et al. 2004). After initial treatment, the recurrence rate in patients is up to 80% and 10-25% of patients could progress to a muscle-invading bladder cancer (Herr 1992). The normal treatment for muscle-invading bladder cancer can be divided into bladder sparing and nonbladder sparing strategies. There is a need for improvement of patient prognosis as about 50% to 75% of patients only survive for 5 years (Stein et al. 2001). Therefore, it is urgent to find a new way to treat bladder cancer in combination with the currently available treatments.

Traditional Chinese medicine (TCM) is widely used in China. Qici Sanling decoction (QCSL), a TCM, is made of the radix of *Astragalus propinquus* Schischkin (Fabaceae) [huangqi], the corm of *Sagittaria sagittifolia* L. (Alismataceae) [cigu], the sclerotium of *Polyporus umbellatus* (Pers.) Fr. (Polyporaceae) [zhuling], the sclerotium of *Poria cocos* (Schw.) Wolf (Polyporaceae) [fuling], the radix of *Paeonia lactiflora* Pall. (Paeoniaceae) [baishao], the radix of *Curcuma zedoaria* (Christm.) Rosc. (Zingiberaceae) [ezhu], the twig of *Cinnamomum cassia* (L.) J.Presl Blume (Lauraceae) [guizhi], the radix of *Glycyrrhiza* glabra L. (Fabaceae) [gancao], the radix of *Rehmannia glutinosa* (Gaertn.) DC (Orobanchaceae) [shudi], and the rhizome of *Smilax glabra* Roxb. (Smilacaceae) [tufuling] at a ratio of 10:10:5:5:5:5:3:2:5:5 in dry weight. In our previous study (Yu et al. 2018), we found that QCSL combined with pirarubicin for treatment of postoperative patients with non-muscle invasion bladder cancer could significantly reduce the cancer recurrence rate, enhance the systemic immunity, and alleviate the side effects caused by chemotherapy drugs.

*Astragalus* root is one of the most popular Chinese herbs, commonly referred to as Radix Astragali, or huang qi. *Astragalus* root is widely used in many TCM preparations and participates in various biological functions (Fu et al. 2014; Li et al. 2014). The main contribution is to counteract the side effects of chemotherapeutic drugs by its immunopotentiating properties and anticancer activity (Zee-Cheng 1992; Ma et al. 2002; Cui et al. 2003; Shao et al. 2004; Zhao LH et al. 2011).

*Sagittaria sagittifolia* is an aquatic flowering plant, with the common name ‘arrowhead’. It is a kind of food that also can be used as medicine. The water extract from *Sagittaria sagittifolia* was reported to exert a hepatoprotective effect in isoniazid and rifampin induced liver injury *in vitro* and *in vivo* (Li et al. 2016; Wang et al. 2018).

In this study, we aimed to explore the possibility of QCSL to treat bladder cancer based on its immune potentiating properties and anticancer activity. This study will provide us with new insights into bladder cancer and identify potential approaches to treat this disease.

## Materials and methods

### T24 human bladder cancer cell culture

T24 human bladder cancer cells were obtained from Cell Bank of the Chinese Academy of Sciences (Shanghai, China) and cultured in RPMI-1640 medium containing 10% FBS, penicillin (100 U/mL) and streptomycin (100 µg/mL) at 37 °C in a 5% CO_2_ humidified incubator.

### Preparation of QCSL and XAV-939

QCSL was purchased from E-Fong pharmaceutical (Guangdong, China) and dissolved with PBS as previously described (Yu et al. 2018). XAV-939, an inhibitor of the WNT/β-catenin pathway, was purchased from Selleck (Houston, TX, USA).

### Mice and nude mouse xenograft model

BALB/c-nu nude mice were purchased from the Shanghai Laboratory Animal Research Centre (Shanghai, China). All mice were 4–6 weeks old and housed at 20–23 °C in a 12 h light/dark cycle with food and tap water supplied *ad libitum*. Nude mouse xenograft models were established as previously described (Zhao P et al. 2016). T24 cells were collected and washed twice in PBS, then the cells were resuspended in PBS to a concentration of 2 × 10^7^ cells/mL. Each mouse was subcutaneously injected with 0.2 mL of cell suspension under the right armpit. Tumour growth was monitored by measuring tumour diameters every 3 days. Tumour volume (mm^3^) was calculated using the equation, V = 0.5 (LW2), where L and W are the length and width of the tumour. After almost 2 weeks, 30 mice were randomly divided into five groups (control; 20 mg/kg XAV-939; 100, 200, and 400 mg/kg QCSL) and each group was given different treatments by gavage once a day. XAV-939 treatment was used as positive control and PBS treatment was used as a negative control. After 7 weeks, the mice were anaesthetised by isoflurane and xenografts were excised. Part of xenografts was fixed in 10% (v/v) formalin for pathological and immunohistochemical examination. Part of the xenografts was frozen for real-time PCR and western blot.

For survival experiments, 60 mice were randomly divided into three groups (control; 20 mg/kg XAV-939; and 400 mg/kg QCSL) and treated as described above. All the mice in each group were housed until natural death.

The experimental procedures for nude mouse xenograft models and daily care were approved by the Committee on Ethical Use of Animals of Shanghai University of Traditional Chinese Medicine. All experiments were performed in accordance with the national legislation and with the National Institutes of Health Guidelines regarding the care and use of animals for experimental procedures.

### Real-time PCR

Relative expression of the selected genes was tested using reverse Transcription System (TaKaRa, Dalian, China) and SYBR Green qPCR Mixes (TaKaRa, Dalian, China). The reactions were performed on a 7900 HT Sequence Detection System (Applied Biosystems, USA), under the following procedure: 50 °C for 2 min; 95 °C for 10 min; 95 °C for 15 s, and 60 °C for 1 min with 40 cycles. Relative quantification of the gene expression level was presented using the comparative Ct method (2^-ΔCt^). The primers used are presented below:

**Table:** 

β-catenin:	Primer F	5′-TTTGCTCAACAAAACAAACGTG-3′
	Primer R	5′-CAGATGAAGCCCCAGTGCC-3′
survivin:	Primer F	5′-TACCGCATCGCCACCTTC-3′
	Primer R	5′-CCAAATCAGGCTCGTTCTCG-3′
c-myc:	Primer F	5′-GGACTGTATGTGGAGCGGTTTC-3′
	Primer R	5′-GTCGTTGAGCGGGTAGGG-3′
Cyclin D1:	Primer F	5′-ATGAACTACCTGGACCGCTTCC-3′
	Primer R	5′-CCGCCTCTGGCATTTTGG-3′
GAPDH:	Primer F	5′-CTGCCCAGAACATCATCC-3′
	Primer R	5′-CTCAGATGCCTGCTTCAC-3′

### Western blot

Protein from xenografts was extracted using RIPA protein extract solution (Solarbio, Beijing, China). Protein concentration was determined by bicinchoninic acid protein assay kit (Thermo, USA). Samples were boiled with 4x loading buffer for 5 min. Aliquots of 15 μg of total protein were separated on 10% or 15% polyacrylamide gels. After electrophoresis, the samples were transferred to polyvinylidene difluoride (PVDF) membranes, blocked overnight at 4 °C in 5% nonfat milk/PBS. Membranes were then incubated for 2 h at room temperature with anti-mouse β-catenin (1:1000, Abcam, USA), anti-mouse survivin (1:1000, Abcam, USA), anti-mouse c-myc (1:1000, Abcam, USA), anti-mouse Cyclin D1 (1:800, Abcam, USA) or anti-mouse GAPDH (1:2000, CST, USA). Membranes were washed and incubated for 1 h with horseradish peroxidase (HRP)-conjugated secondary antibodies (Beyotime, Beijing, China), the proteins were visualised using an ECL solution (Millipore, USA) and imaged using a Tanon-5200 Multi system (Tanon, Shanghai, China).

### Histologic examination and TUNEL

After 48 h fixation in 10% (v/v) formalin, tumour tissues obtained from T24 nude mouse xenograft models were paraffin embedded for histologic examination. Sections (5 mm; Leica RM2125, Germany) were stained with haematoxylin and eosin (H&E) according to standard methods. Light microscopy (Olympus, Tokyo, Japan) was used to collect the images at 200× magnification.

For apoptosis detection, sections were stained with reaction buffer applied by a TUNEL Kit (Roche, Indianapolis, IN, USA) following the manufacturer’s instructions. Percentages of apoptotic cells were evaluated in five randomly selected fields.

### Immunohistochemistry

Tumour tissue sections were placed in a pressure cooker for 2 min to perform antigen retrieval and then were incubated with primary antibody (anti-mouse Ki-67, 1:300, Abcam) overnight at 4 °C. The sections were then incubated with HRP conjugated secondary antibody (Beyotime, Beijing, China) for 30 min. 3, 3′-diaminobenzidine (DAB) and haematoxylin were used for developing and counterstaining. Light microscopy (Olympus, Tokyo, Japan) was used to collect the images at 200× magnification.

### Statistical analysis

Data are shown as the mean ± standard deviation and SPSS.20 software (SPSS, Inc., Chicago, IL) was used to evaluated statistics. Student’s *t*-test was used to compare the differences between two groups and *p* < 0.05 was considered statistically significant.

## Results

### QCSL inhibits T24 xenograft growth and increases the survival time of T24 xenograft mice

**Figure 1.**
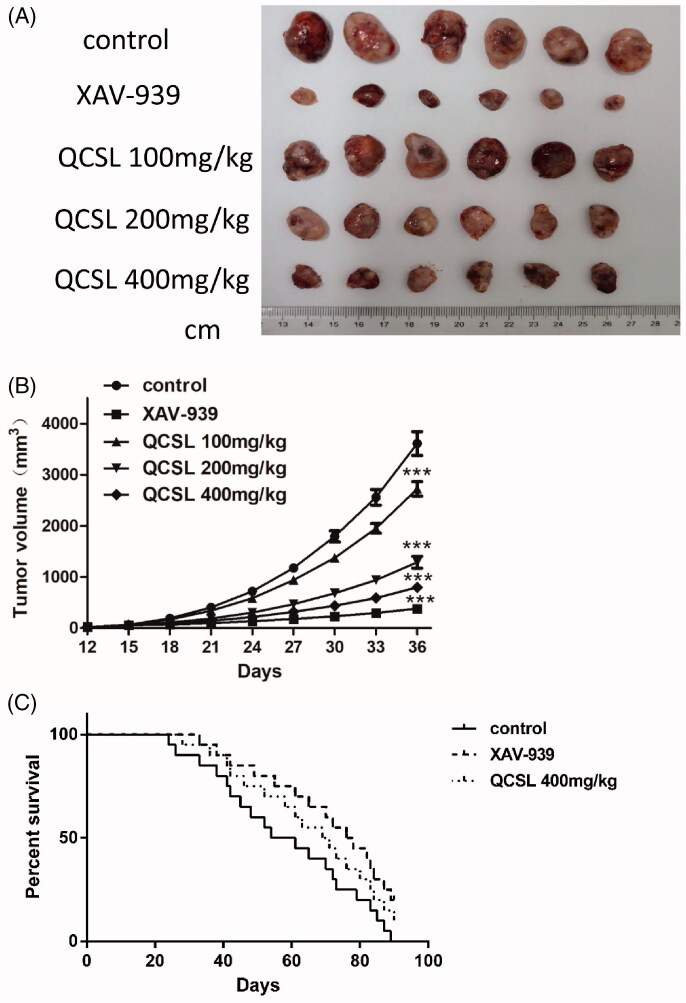
QCSL inhibits the growth of T24 xenografts and increases the survival time of T24 xenograft mice. Thirty mice were randomly divided into five groups (*n* = 6 each): control group; 20 mg/kg XAV-939 group; 100 mg/kg QCSL group; 200 mg/kg QCSL group and 400 mg/kg QCSL group. (A) Images of T24 xenografts treated with 20 mg/kg XAV-939 or different concentrations of QCSL for 20 days. (B) The volume of T24 xenografts in different groups. (C) Changes in survival time during treatment with XAV-939 or QCSL. ***p < 0.001 compared to control group.

In order to evaluate the antitumor effect of QCSL *in vivo*, T24 xenografts were performed. When T24 xenografts grew to a visible size, 30 mice were randomly divided into five groups (*n* = 6 each) and treated as previously described. As shown in Figure 1, treatment with either XAV-939 or QCSL caused reductions in tumour volume compared to the control group. Treatment with XAV-939 showed the best inhibition of tumour growth. The results also showed a dose-dependent inhibition of tumour growth when treated with 100, 200 and 400 mg/kg QCSL compared to the control group (*p* < 0.001). Furthermore, we found the survival time in the XAV-939 and 400 mg/kg QCSL treated groups to be extended as compared with the control group. These data indicate that QCSL inhibits the growth of T24 xenografts and increases the survival time of T24 xenograft mice.

### Histological examination of T24 xenografts

**Figure 2.**
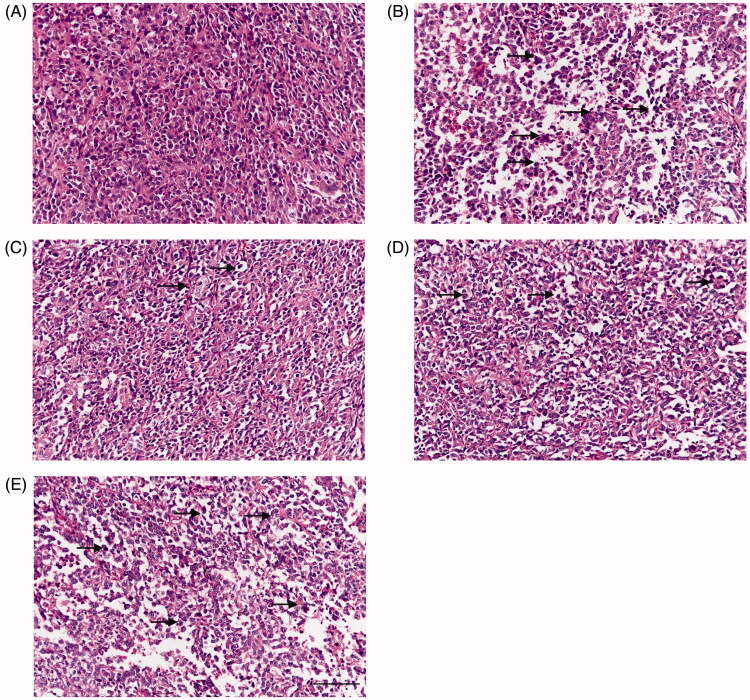
Histological examination of T24 xenografts (H&E staining). (A) Control group; (B) 20 mg/kg XAV-939 group; (C) 100 mg/kg QCSL group; (D) 200 mg/kg QCSL group; (E) 400 mg/kg QCSL group. Scale bars: 100 μm.

H&E staining is a regular way to explore disease pathological state. After sacrificing the mice, T24 xenografts were collected for H&E staining. As expected, irregular shapes and heterologous, sizes of cells as well as mitotic cells were observed (Figure 2). Therefore, the tumour tissues were confirmed as cancer tissues. Some inflammatory cells and nuclear pyknotic cells were observed in the 100 and 200 mg/kg QCSL treatment groups. Even more inflammatory cells and nuclear pyknotic cells were seen in 400 mg/kg QCSL and XAV-939 treatment group.

### QCSL induces the apoptosis of T24 xenografts

**Figure 3.**
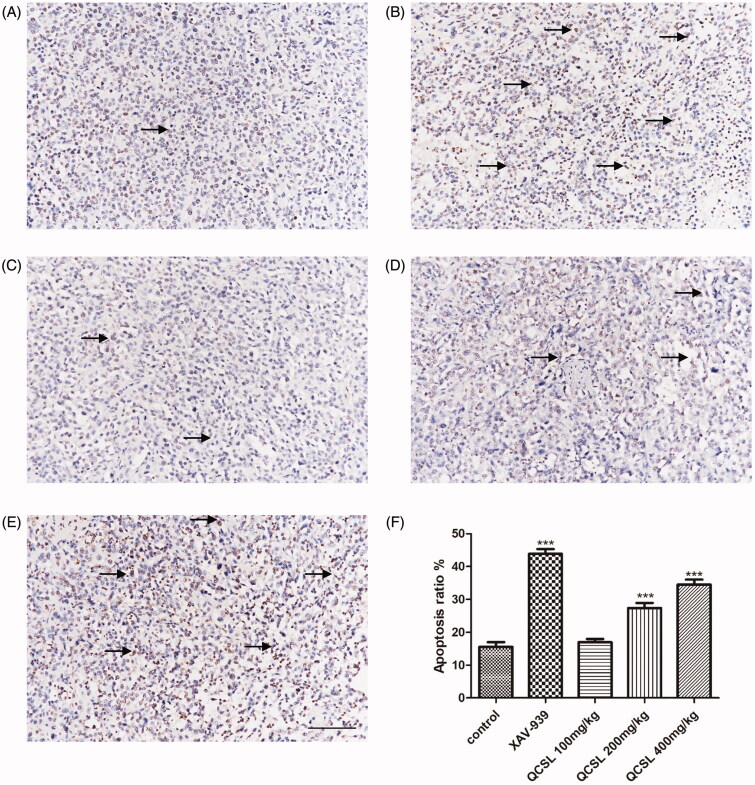
TUNEL staining of T24 xenografts. (A) Control group; (B) 20 mg/kg XAV-939 group; (C) 100 mg/kg QCSL group; (D) 200 mg/kg QCSL group; (E) 400 mg/kg QCSL group; (F) Apoptosis rates of groups with different treatments. ***p < 0.001 compared to control group. Scale bars: 100 μm.

TUNEL staining was used to determine the percentage of apoptotic cells in the five groups. As shown in Figure 3, a few positive cells were found in the control group and 100 mg/kg QCSL group, while many positive cells were found in the 200 and 400 mg/kg QCSL treatment groups. Treatment with XAV-939 showed the most positive cells. These results suggested that QCSL induced the apoptosis of T24 xenografts.

### QCSL inhibits cell proliferation *in vivo*

Based on previous results, we found QCSL could inhibit the growth of T24 xenografts. Thus, we detected cell proliferation of cells using proliferative marker Ki-67 in the xenograft tumours. Immunohistochemistry results showed that treatment with either XAV-939 or QCSL caused a decrease of Ki-67 positive cells compared to the control group. Treatment with XAV-939 showed the lowest number of Ki-67 positive cells. The results also showed a dose-dependent decrease of Ki-67 positive cells in the groups that were treated with 100, 200, and 400 mg/kg QCSL. These data suggested that QCSL inhibits the expression level of Ki-67 in T24 xenografts.

### QCSL inhibits tumour growth via regulating WNT/β-catenin pathway

**Figure 4.**
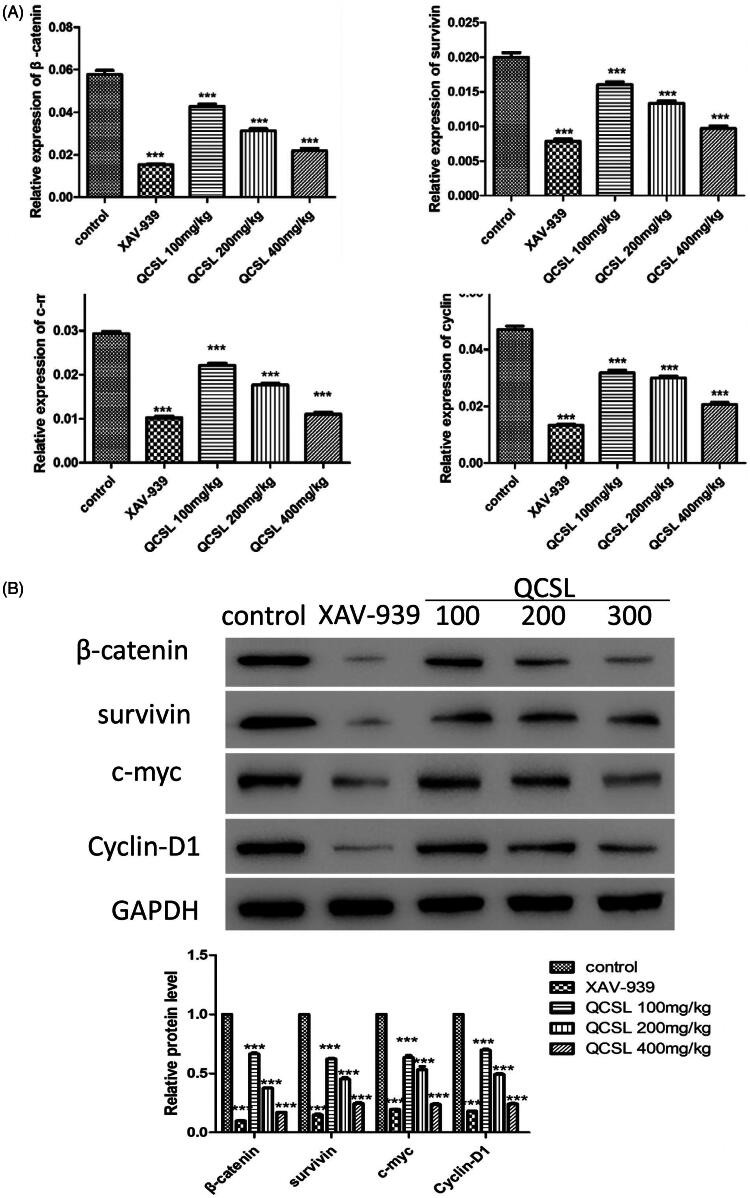
QCSL inhibits tumour growth via regulation of the WNT/β-catenin pathway. (A) The mRNA expression levels of β-catenin, survivin, c-myc and cyclin D1 were measured by real-time PCR. (B) The protein expression levels of β-catenin, survivin, c-myc and cyclin D1 were analysed by western blot. ***p < 0.001 compared to control group.

To further explore the mechanism of QCSL inhibition of tumour growth, we measured the expression level of markers of the WNT/β-catenin pathway. As shown in Figure 4, the mRNA and protein expression level of β-catenin, survivin, c-myc, and cyclin-D1 were inhibited after treatment with either XAV-939 or QCSL compared to the control group. The lowest expression levels of these proteins were found in the XAV-939 group. We also observed a dose-dependent inhibition of these proteins in the groups treated with 100, 200, and 400 mg/kg QCSL compared to the control group (*p* < 0.001). These data suggest that QCSL inhibits tumour growth via regulation of the WNT/β-catenin pathway.

## Discussion

It has been reported that β-catenin plays a central role in cell adhesion (McCrea et al. 1991). Other studies have revealed a regulatory role of β-catenin in contributing to the transmission of proliferation signals to the WNT/T-cell factor pathway (Miller and Moon 1996; Peifer 1997). Usually, β-catenin is located in the cell membrane to carry out its cell adhesion functions. The function of β-catenin in the cytoplasm and nucleus reflects its role as a mediator of WNT/β-catenin signalling (Barth et al. 1997). C-myc (He et al. 1998) and cyclin D1 were reported as putative target genes of WNT signalling. In this study, to test the effects of QCSL on WNT/β-catenin signalling, we immunoblotted for components of the pathway and found that increased doses of QCSL dramatically inhibited β-catenin, survivin, c-myc and cyclin D1 (Figure 4). As reported, survivin is upregulated in almost all human tumours including bladder cancer (Ambrosini et al. 1997; Fukuda and Pelus 2006). Survivin could be regulated by Wnt signalling and may play an antagonist role in cancer stem cells (Reya et al. 2001).

**Figure 5.**
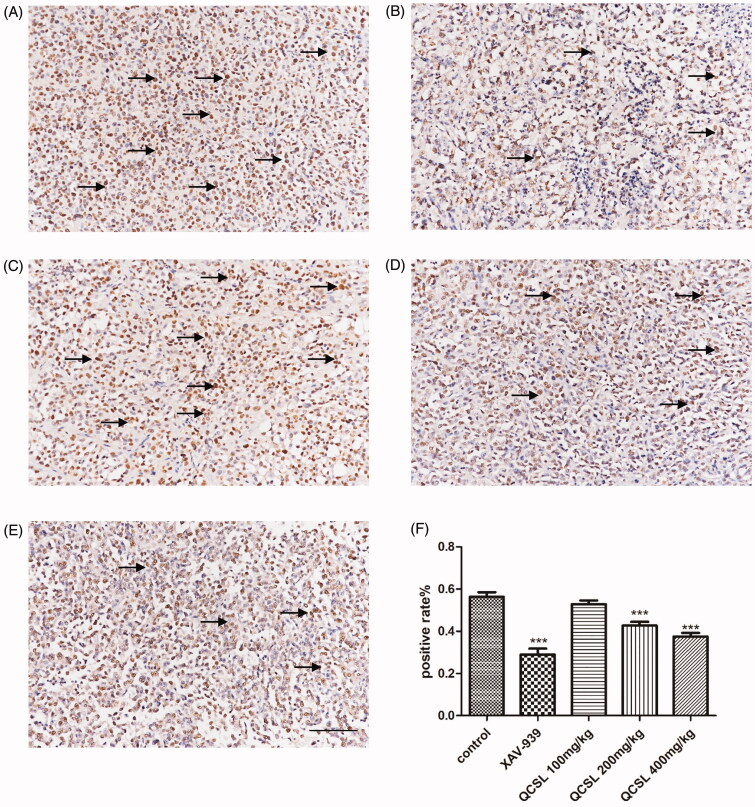
Representative image of Ki-67 staining in xenograft tumour sections. (A) Control group; (B) 20 mg/kg XAV-939 group; (C) 100 mg/kg QCSL group; (D) 200 mg/kg QCSL group; (E) 400 mg/kg QCSL group. (F) Ki-67 positive rates of groups with different treatments. ***p < 0.001 compared to control group. Scale bars: 100 μm.

C-myc and cyclin D1 are reported to induce S phase entry and cell-cycle progression, in parallel, as well as contribute to the neoplastic transformation of cells (Haas et al. 1997). The transcription factor, T-cell factor 4, can bind to the promoter of c-myc, and cyclin D1 and these two genes could be targets of the WNT/β-catenin signalling pathway (Brabletz et al. 1999; Zhang et al. 2018). The expression of myc protein has a relationship with cell proliferation in bladder cancer (Lipponen 1995). To test the effects of QCSL on tumour growth, we treated mice carrying T24 derived xenograft tumours with XAV-939 or QCSL. We found that XAV-939 and QCSL can dramatically inhibit tumour growth (Figure 1). Furthermore, TUNEL staining and Ki-67 staining revealed that XAV-939 and QCSL treatment induced apoptosis and inhibited cell proliferation (Figures 3 and 5) and that this is due to regulation of the WNT/β-catenin target genes survivin, c-myc and cyclin D1 (Figure 4).

## Conclusions

In this study, we showed primary data to support that QCSL can dramatically suppress tumour growth by inhibiting the WNT/β-catenin pathway, which could be validated by XAV-939 treatment. The long term goal of this study is to test the possibility of QCSL treatment of bladder cancer in the future. For this purpose, further mechanistic studies are needed. As ligands such as wnt3, wnt5 and CBP play important roles in activation of the WNT/β-catenin pathway, we still need to explore how QCSL affects the activation of the pathway. On the other hand, we need to expand our research to clinical specimens to test potential clinical effects on bladder cancer or to predict potential biomarkers for classifying patients before standard treatments.
